# Associations between Maternal Health-Related Quality of Life during Pregnancy and Birth Outcomes: The Generation R Study

**DOI:** 10.3390/ijerph16214243

**Published:** 2019-11-01

**Authors:** Guannan Bai, Ida J Korfage, Eva Mautner, Hein Raat

**Affiliations:** 1 Department of Public Health, Erasmus MC-University Medical Centre Rotterdam, Wytemaweg 80, Rotterdam, 3015 CN South Holland, The Netherlands; g.bai@erasmusmc.nl (G.B.); i.korfage@erasmusmc.nl (I.J.K.); 2 The Generation R Group, Erasmus MC- University Medical Centre Rotterdam, Wytemaweg 80, Rotterdam, 3015 CN South Holland, The Netherlands; 3 Department of Obstetrics and Gynecology, Medical University of Graz, Auenbruggerpl. 2, Graz 8036, Austria; Eva.Mautner@gmx.at

**Keywords:** birth outcomes, pregnancy, quality of life

## Abstract

The objective of this study was to assess associations between maternal health-related quality of life (HRQoL) in early, mid-, and late pregnancy and birth outcomes and to assess the differences in birth outcomes between subgroups of mothers reporting relatively “low” and relatively “high” HRQoL. HRQoL was measured by the 12-item Short Form Health Survey in early (n = 6334), mid- (n = 6204), and late pregnancy (n = 6048) in a population-based mother and child cohort; Physical and Mental Component Summary (PCS/MCS) scores were calculated. Birth outcomes included pregnancy duration, preterm birth, birth weight, low birth weight, and small for gestational age. We defined very high PCS/MCS scores as the >90th percentile and very low score as the <10th percentile. The lower PCS score in late pregnancy was significantly associated with a higher chance of having small-for-gestational-age birth (per 10 points: OR = 1.20, 95% CI: 1.08, 1.33, *p* value = 0.0006). In early, mid-, and late pregnancy, the subgroup mothers with a low MCS score had infants with a lower average birth weight than those with very high scores (*p* < 0.05). The association between higher physical HRQoL in late pregnancy and a higher chance of having small-for-gestational-age birth needs further research. The role of mother’s mental HRQoL during pregnancy and the potential consequences for the child require further study.

## 1. Introduction

Preterm birth, small-for-gestational-age birth, and low birth weight are relevant indicators for new-born mortality and morbidity [1,2,3]. Health impairments due to these adverse birth outcomes may last until adulthood [3,4]. Maternal health factors are associated with adverse birth outcomes; examples are maternal lifestyle-related factors (e.g., tobacco/alcohol use and body mass index) and medical conditions during pregnancy (e.g., preeclampsia, diabetes, and depression) [5,6]. Therefore, it is plausible that indicators of the overall maternal health during pregnancy, for example, health-related quality of life (HRQoL), may be associated with birth outcomes.

HRQoL is a measure of the personal perception of the quality and value of life in the context of impairments, functional states, and opportunities as influenced by disease, injury, treatment, and policy [7]. The rating of HRQoL could be used in health care as a tool for identifying patients who are in need of additional care. For example, in the adult population, a relatively low level of HRQoL has been shown to be predictive of short- and long-term hospitalization, morbidity. and mortality [8,9,10,11]. However, data on the associations between maternal HRQoL during pregnancy and normal or adverse birth outcomes are scarce. 

To our knowledge, only two relevant studies were conducted [12,13]. A study in Austria among 90 women showed that those who gave birth to a preterm infants had reported worse physical HRQoL during pregnancy than those who gave birth to a term infant [12]. This study concluded that relatively low HRQoL during pregnancy was associated with the risk for preterm delivery. The other study among 198 women in Hong Kong, China, demonstrated associations between three domains of HRQoL (i.e., physical, mental, and social) during pregnancy and preterm birth and low birth weight. The authors concluded that poor HRQoL in late pregnancy can predict preterm birth [13]. 

Given the limited number of relevant studies, the present study aimed to investigate the associations between maternal physical and mental HRQoL in early, mid-, and late pregnancy and birth outcomes in a large prospective population-based mother and child cohort, the Generation R study [14]. Our hypothesis is that low (physical/mental) HRQoL is associated with adverse birth outcomes.

In the Generation R Study, the participants are relatively healthy and they are expected to have a relatively high HRQoL [15,16]; therefore, we will additionally explore the differences in birth outcomes between the subgroups of pregnant women who reported very low (<10th percentile) and very high (>90th percentile) (physical/mental) HRQoL scores. Our hypothesis is that the subgroup of pregnant women reporting very low HRQoL during pregnancy have, on average, worse birth outcomes compared to the birth outcomes in the subgroup of women reporting very high HRQoL during pregnancy. 

## 2. Materials and Methods 

### 2.1. Data Source

The present study was embedded in a prospective population-based mother and child cohort from fetal life until adulthood in the Netherlands. Briefly, the cohort includes 9778 mothers living in the Rotterdam area and their children born between April 2002 and January 2006. The response rate was approximately 61%. Although the cohort aimed to enroll women in early pregnancy (gestational age < 18 weeks) when it was set up, enrolment was possible until the postnatal period: 7069 mothers were enrolled in early pregnancy, 1594 were enrolled in mid-pregnancy (gestational age 18−25 weeks), 216 were enrolled in late pregnancy (gestational age ≥25 weeks), and 899 were enrolled in postpartum phase. This cohort study has been described previously in detail [14,15]. The study was approved by the Medical Ethical Committee of the Erasmus MC-University Medical Center Rotterdam [16]. The approval number is 217.595/2002/203. The date of approval is 9 January 2003. Written consent had been obtained from all participating women.

### 2.2. Study Population

**Figure 1:**
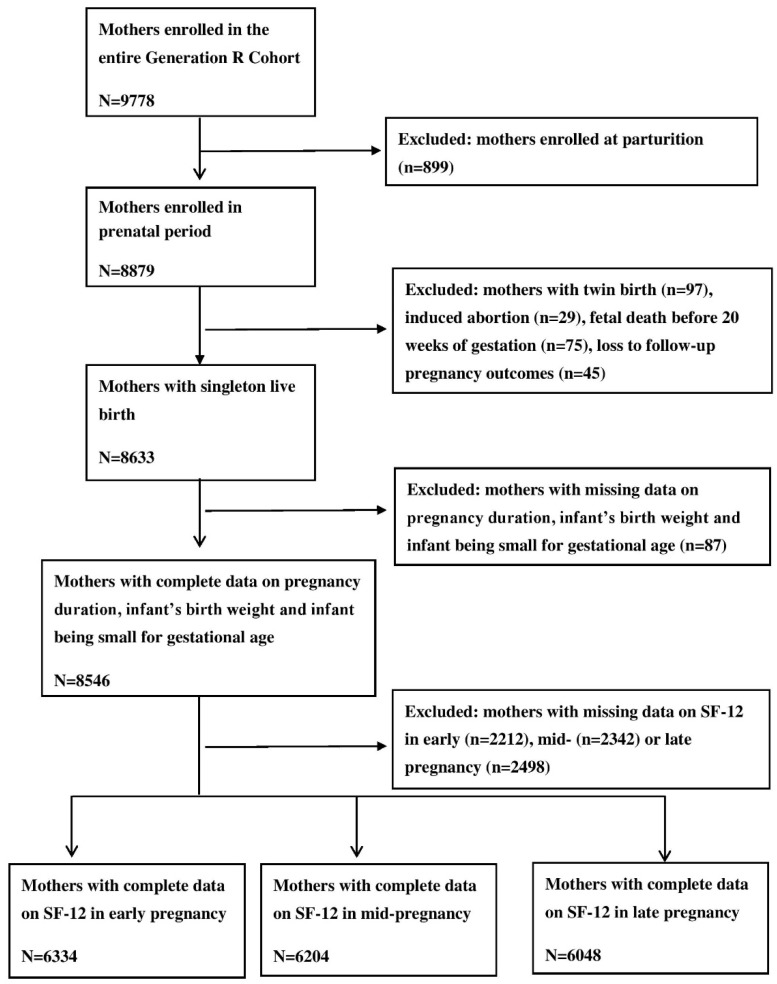
Flow chart of the study populations.

Of the 9778 women who were enrolled in the cohort study, 8879 women enrolled during the prenatal period. We excluded women with pregnancies with the following outcomes: twin birth (n = 97), induced abortion (n = 29), fetal deaths before 20 weeks of gestation (n = 75), and loss of follow-up in the prenatal period (n = 45). Additionally, we excluded women with missing data on pregnancy duration, infant’s birth weight, and the infant being small for gestational age (n = 87). Further, we excluded women with missing data on one or more 12-item Short Form Survey (SF-12) items in early (n = 2212), mid- (n = 2342), and/or late pregnancy (n = 2498). Thus, data of 6334 women in early pregnancy (dataset 1), of 6204 women in mid-pregnancy (dataset 2), and of 6048 women in late pregnancy (dataset 3) were included in the analyses (See Figure 1).

### 2.3. HRQoL

HRQoL was measured using the 12-item Short Form Survey (SF-12) in early, mid-, and late pregnancy. It is a reliable and well-validated instrument to measure HRQoL and is widely used in studies with large sample sizes [17]. SF-12 consists of 12 items that covers eight areas: physical functioning, role limitations due to physical problems, bodily pain, general health, vitality, social functioning, role limitation due to emotional problems, and perceived mental health. Two component summary scores were yielded: the Physical and the Mental Component Summary (PCS/MCS) scores [18]. A higher score indicates better HRQoL.

### 2.4. Birth Outcomes

Information on pregnancy duration (weeks) and birth weight (grams) was obtained from patient records as completed by community midwives and obstetricians. Preterm birth was defined as the birth of an infant before 37.0 weeks of gestation [1] and low birth weight was defined as a birth weight <2500 grams [19]. In this study, being small for gestational age was defined as a birth weight below the 10th percentile for gestational age and based on standard deviation curves derived from this cohort [20].

### 2.5. Covariates

Based on a review of the literature [21,22,23,24,25,26], we selected the following potential confounders that were available in our dataset and included them in the regression analyses: maternal age at enrolment, gestational age at enrolment, parity, ethnic background (native Dutch, other Western immigrant, and non-Western immigrant), educational level (low, mid-low, mid-high, and high), body mass index at enrolment, and maternal smoking measured in each gestational period. Maternal ethnic background was defined according to the classification of Statistics Netherlands [27]. According to Dutch Standard Classification of Education, we generated four categories of education in our study: high (university degree), mid-high (higher vocational training and bachelor’s degree), mid-low (>3 years general secondary school and intermediate vocational training), and low (no education, primary school, lower vocational training, intermediate general school, or 3 years or less general secondary school) [28]. Maternal smoking in pregnancy was measured in early, mid-, and late pregnancy by self-report questionnaires. In early pregnancy, women were asked “Have you smoked in the past three months?” with three answer options: “never”, “stopped when pregnancy was known”, and “continued with smoking during pregnancy”. In mid- and late pregnancy, women were asked “Have you smoked in the past three months?” with two answer options: “yes” and “no”.

### 2.6. Statistical Analyses

Descriptive analyses were applied to characterize women enrolled in early pregnancy (n = 6334). Supplementary Table S1 shows the results of this nonresponse analysis. Women who were excluded from the analyses were more often non-Dutch/non-Western, more often had low education, had higher body mass index, more often continued smoking in pregnancy, and had higher incidences of adverse birth outcomes (i.e., preterm birth, low birth weight, and small for gestational age birth) (*p* < 0.05).

We applied multivariate linear regression analysis (for continuous outcome variables) and logistic regression model (for categorical outcome variables) to assess the associations between physical/mental component summary score in early, mid-, and late pregnancy and birth outcomes. In these models, we recoded the original physical/mental component summary score, i.e., we divided by 10, so, in the regression models, 1 point reflects 10 points of the original physical and mental component summary score following the approach proposed by Mapes et al [9]. The regression models were adjusted by the covariates.

Differences in birth weight and pregnancy duration were assessed between subgroups of women reporting very low (<10th percentile) and very high (>90th percentile) physical and mental component summary scores using two independent sample t-tests. Cohen’s effect sizes (d) were calculated by dividing the difference in mean scores among subgroups by largest SD and interpreted as 0.2 ≤ d < 0.5 small difference, 0.5 ≤ d < 0.8 moderate difference, and d ≥0.8 large difference [29]. Differences in the incidence of infants of low birth weight, preterm birth, and being small for gestational age were assessed between subgroups of women reporting very low (<10th percentile) and very high (>90th percentile) physical/mental component summary scores using chi square tests.

Because we have conducted multiple analyses with the dependent variable, a Bonferroni correction was conducted. The Bonferroni-corrected *p* value was calculated by dividing the original *p* value (*p* = 0.05) by the number of analyses with the dependent variable, i.e., *p* corrected = 0.05/6 = 0.008. In our study, *p* < 0.008 indicated statistical significance.

We conducted all analyses with the Statistical Package for Social Sciences (SPSS) version 21.0 for Windows (IBM Corp., Armonk, NY, USA).

## 3. Results

### 3.1. General Characterisitics of Mothers and Children

Table 1 presents the characteristics of women enrolled in early pregnancy. The mean maternal age is 29.9 years (SD 5.2). The mean gestational age at intake was 15 weeks (SD 4.0); 3679 (58.2%) of women had their first pregnancy; 3375 (53.5%) of women were Dutch; 1563 (25.1%) and 1234 (19.8%) had high or mid-high educational level; and the mean BMI at enrolment was 24.7 (SD 4.5). In early pregnancy, 4738 (75.9%) of women had never smoked; 761 (12.2%) stopped smoking when the pregnancy became known; and 746 (11.9%) continued to smoke during pregnancy. In mid-pregnancy, 935 (15.4%) of women had smoked in the previous three months (data not shown). In late pregnancy, 891 (14.9%) of women had smoked in the previous three months (data not shown). The average physical component summary scores as reported in early, mid-, and late pregnancy were 47.6 (SD 9.1) (see Table 1), 46.2 (SD 9.5), and 39.0 (SD 9.1), while the average mental component summary scores as reported in early, mid-, and late pregnancy were 48.7 (SD 10.4) (see Table 1), 51.3 (SD 9.7), and 54.1 (SD 10.4). The mean pregnancy duration was 39.9 weeks (SD 1.7); 331 (5.2%) of women had preterm infants. Mean birth weight was 3428 grams (SD 558); 290 (4.6%) of women had infants with low birth weight; and 604 (9.5%) of women had infants who were small for gestational age.

### 3.2. Associations between Physical and Mental HRQoL during Pregnancy and Birth Outcomes

Table 2 presents the associations between physical and mental HRQoL scores in each gestational period and birth outcomes adjusted by the covariates (maternal age at enrolment, gestational age at enrolment, maternal educational level, maternal ethnic background, BMI at enrolment, and maternal smoking in each gestational period). Applying the adjusted significance level, a ten-point increase in physical component summary score in late pregnancy was statistically significantly associated with a higher chance of having small-for-gestational-age birth (OR = 1.20, 95% CI:1.08, 1.33, *p* value = 0.0006). No other significant associations between physical/mental component summary score in pregnancy and birth outcomes were found.

### 3.3. Differences in Birth Outcomes between Subgroups with Very High vs. Very Low HRQoL during Pregnancy

Table 3 shows differences in birth outcomes between subgroups reporting very high (>90th percentile) and very low (<10th percentile) physical/mental component summary score in early, mid-, and late pregnancy.

According to the Bonferroni-corrected *p* value, the average pregnancy duration, the average birth weight, the incidence of preterm birth, and low birth weight did not significantly differ between subgroups of women reporting a low physical component summary score and the subgroup reporting a high score in early, mid-, and late pregnancy (*p* values >0.008). Having a small-for-gestational-age birth was less frequent in the subgroup mothers who reported a low physical component summary score compared to the subgroup mothers who reported a high score in late pregnancy (7.5% vs. 12.3%, *p* = 0.005). The average pregnancy duration, the occurrence of preterm birth, and low birth weight did not differ between subgroups of women reporting a low mental component summary score compared to women reporting a high score in early, mid-, and late pregnancy (*p* values >0.008). The average birth weight of infants whose mothers reported a low mental component summary score was significantly lower than that of infants whose mothers reported a high score (early pregnancy: 3351 vs. 3440 grams, *p* = 0.005, d = 0.15; mid-pregnancy: 3376 vs. 3474 grams, *p* = 0.001, d = 0.18; and late pregnancy: 3344 vs. 3474 grams, *p* < 0.001, d = 0.24). The occurrence of having a small-for-gestational-age birth was significantly higher in the subgroup mothers reporting a low mental component summary score compared with the subgroup mothers reporting a high score in late pregnancy (12.3% vs. 6.4%, *p* = 0.001).

## 4. Discussion

Our study explored the associations between women’s physical and mental HRQoL in each gestational period and birth outcomes. We found a significant association between better physical HRQoL in late pregnancy and higher chances of having a small-for-gestational-age birth. In addition, we found statistically significant differences in several birth outcomes between the subgroups of women reporting relatively high and relatively low HRQoL in pregnancy, but Cohen’s effect sizes were small.

Our study did not confirm the hypothesis that worse physical HRQoL in early, mid-, and late pregnancy is associated with preterm birth, gestational duration, and (lower) birth weight. This may be because relatively healthy women were enrolled in the Generation R Study; participants were relatively often highly educated and healthy and had a relatively highly HRQoL compared to clinical study samples and compared to the general population [25,26,30]. Therefore, the number of infants with clinically severe outcomes, such as early/moderate preterm birth and very low birth weight, was relatively low. This limits the power to detect significant associations between HRQoL during pregnancy and adverse birth outcomes. Therefore, we recommend evaluating mothers’ HRQoL during pregnancy and the associations with birth outcomes in other large and varied community samples and in clinical samples. Such studies may enhance our understanding of the associations between mothers’ HRQoL in pregnancy and birth outcomes.

In contrast with the findings by Mautner et al. [12] and by Wang et al. [13], in the Generation R study, in late pregnancy only, an increase in physical HRQoL was associated with more frequent small-for gestational-age births. In addition, also in contrast with the findings by Mautner et al. [12] and by Wang et al. [13], in the Generation R study, in the subgroup of women reporting relatively low HRQoL in late pregnancy, the occurrence of having a small-for-gestational-age birth was significantly lower than in the subgroup of women reporting relatively high physical HRQoL. Further, we explored whether this significant difference remained after including potential confounders in the multivariate regression model. We found this difference still remained statistically significant (see Supplementary Table S2). In addition, we assessed additional factors that might confound the association, such as the presence of pregnancy-related conditions (preeclampsia, gestational diabetes, and pregnancy-induced hypertension), presence of maternal psychopathology during pregnancy, mother’s height, and the presence of obesity before pregnancy. After including these variables in the regression model, the association between higher physical HRQoL in late pregnancy and occurrence of small-for-gestational-age (SGA) birth remained statistically significant (data not shown). Therefore, in our study, the abovementioned factors did not explain the association between (higher) physical HRQoL in late pregnancy and SGA. Regarding this association, a potential explanation may be that women who will give birth to infants with a relatively small size may possibly gain less weight during pregnancy, which gave less burden on their own physical health in the last phase of their pregnancy, so these women may perceive relatively better physical HRQoL [31]. We explored this in our data and found that women who had SGA offspring more often had less weight gain (i.e., “inadequate weight gain” according to the Institute of Medicine guideline) during pregnancy compared to women who did not have SGA offspring (32% versus 18%, *p* < 0.001). In our study, fetal growth was measured by weight; however, weight is the result of multiple phenomena including lean mass growth, length growth, and increase of head circumference. Therefore, we additionally repeated the analyses in Table 2 and Table 3 for offspring length and head circumference at birth in order to explore whether length and head circumference growth could provide insight into the association between better physical HRQoL in late pregnancy and relatively more SGA infants. The results are presented in Supplementary Tables S3–S5. After including potential confounders in the models, there were no associations between HRQoL during pregnancy and offspring length and head circumference at birth. Therefore, in our study, this did not seem to provide information explaining the association of higher physical HRQoL score assessed in late pregnancy and higher prevalence of SGA infants. Lastly, we would like to note that women who perceived their health status as “physically healthy” (as indicated by relatively high PCS scores) in late pregnancy may have a different, healthier lifestyle than those who perceived their physical health status as relatively poor (as indicated by relatively low PCS scores). In this study, we did not explore the influence of lifestyle on the associations between HRQoL and child birth outcomes. Therefore, we recommend investigating this issue in future studies.

In the whole Generation R study sample, our findings did not confirm the hypotheses that worse mental HRQoL in early, mid-, and late pregnancy is associated with more preterm birth, shorter gestational duration, lower birth weight, and more often small-for-gestational-age birth. This is in contrast with the results of the study by Wang et al. that showed that women reporting better mental health in pregnancy (25−29 weeks) had a lower risk of having low-birth-weight infants; we did not replicate that finding in the analyses in the total sample [13].

However, the subgroup of women reporting relatively low mental HRQoL during pregnancy in the Generation R study had infants with a lower average birth weight in comparison with the subgroup of women reporting the relatively high mental HRQoL. This confirms the abovementioned finding by Wang et al. [13]. However, the effect sizes were small. We also found a higher incidence of having a small-for-gestational-age birth in the subgroup of women who reported relatively low mental HRQoL in early, mid-, and late pregnancy compared with the subgroup who reported relatively high mental HRQoL. The lower level of mental HRQoL during pregnancy may be related to a worse maternal psychosocial health status. This, in turn, may be influenced by psychological symptoms and disorders, for instance, maternal depressive symptoms and depression that have been reported by approximately 20% of pregnant women [32]. Depression is known to be related to impaired fetal growth [32,33,34,35,36,37]. However, it might also be the case that the results from antenatal examinations may inform mothers that their infant might be at risk for having lower weight or becoming small for gestational age; this may have affected mother’s mental HRQoL in a negative way. We additionally explored whether the abovementioned four significant differences remained after correcting for the potential confounders. However, we did not confirm the significant associations of mental HRQoL scores in early, mid-, and late pregnancy with birth weight and the association of mental HRQoL scores in late pregnancy with small–for-gestational-age birth between the subgroup of women reporting very high (>90th percentile) and the subgroup of women reporting very low (<10th percentile) scores (see Supplementary Table S2).Therefore, we recommend further studies in other populations to confirm or reject our findings.

This is one of the few studies regarding the association between women’s HRQoL during pregnancy and birth outcomes. The present study was embedded in large prospective population-based mother and cohort study, which enabled a large sample size for the analyses. Data on more than 6000 women in early, mid-, and late pregnancy was available. To prevent collinearity that was observed in an earlier study [13], we analyzed the associations of HRQoL in each gestational period separately with birth outcomes.

There are some limitations that we need to acknowledge. First, the exclusion of participants with missing data may limit the generalizability of results from the population for analysis to some extent. As shown by the nonresponse analysis, women included in the present study were more often relatively highly educated, more often Dutch, and less often had adverse birth outcomes compared to women excluded from analyses. Therefore, the results should be interpreted with caution; also, because of this, there may be an underestimation of the strength of the associations between maternal HRQoL during pregnancy and birth outcomes. Second, restricted growth or risk factors thereof may appear already at the beginning of the pregnancy. Therefore, we propose to measure women’s HRQoL before pregnancy in future cohort studies of parents who anticipate having children, such as the Generation R Next [38]. Third, we could not include all potential confounding variables in the analyses, for example, diet-related factors. As suggested by previous studies, healthy dietary patterns may be associated with a lower risk of adverse birth outcomes [39]. David Ruth et al. have shown that a higher “protein diet” pattern during pregnancy may be associated with a higher risk of having SGA offspring and that a higher risk of neonatal deaths in an underprivileged urban population in the United States [40]. However, there is also evidence showing that, among relatively well-nourished women in the industrialized countries, the impact of diet on birth outcomes seems marginal [41]. In this study, we did not assess the diet patterns during pregnancy. Therefore, we recommend evaluating the role of diet pattern when assessing the association between maternal HRQoL and birth outcomes in future studies.

## 5. Conclusions

In the total study population, our findings did not confirm the hypotheses that low maternal physical and mental HRQoL in early, mid-, and late pregnancy is associated with more preterm birth, shorter pregnancy duration, and lower birth weight. In contrast, in late pregnancy, we saw that a relatively better physical HRQoL was associated with a higher chance of having a small-for-gestational-age birth. This requires further study. Our study showed small effects regarding a relatively low average birth weight and more frequent small-for-gestational-age birth in the subgroup with a relatively low mental HRQoL compared with the subgroup with a relatively high mental HRQoL. The role of mother’s mental HRQoL during pregnancy and the potential consequences for the child require further study. 

## Figures and Tables

**Table 1:** Characteristics of mothers at baseline and birth outcomes (N = 6334).

Variables.	Values *
Maternal age at enrolment (years), mean (SD)	29.9 (5.2)
Gestational age at enrolment (weeks), mean (SD)	15.0 (4.0)
Parity, number (%)	
Nulliparous	3679 (58.2)
Multiparous	2642 (41.8)
Missing	13
Educational level, number (%)	
High education	1563 (25.1)
Mid-high	1234 (19.8)
Mid-low	1929 (30.9)
Low	1507 (24.2)
Missing	101
Ethnic background, number (%)	
Dutch	3375 (53.5)
Non-Dutch, Western	552 (8.8)
Non-Dutch, non-Western	2378 (37.7)
Missing	29
Body mass index at enrolment, mean (SD)	24.7 (4.5)
Missing	29
Maternal smoking in early pregnancy, number (%)	
Never smoking	4738 (75.9)
Stopped smoking when the pregnancy was known	761 (12.2)
Continuing smoking in pregnancy	746 (11.9)
Missing	89
HRQoL score, SF-12	
Physical component summary score, mean (SD)	47.6 (9.1)
Mental component summary score, mean (SD)	48.7 (10.4)
Birth outcomes	
Pregnancy duration (weeks), number (%)	39.9 (1.7)
Preterm birth (yes), number (%)	331 (5.2)
Birth weight (grams), mean (SD)	3428 (558)
Low birth weight (yes), number (%)	290 (4.6)
Small for gestational age (yes), number (%)	604 (9.5)

* Values in this table are means, standard deviations (SD), numbers, and percentages. HRQOL: Health-related quality of life; SF-12: 12-item Short Form Survey.

**Table 2:** Associations between physical and mental component summary (PCS/MCS) scores in each gestational period and birth outcomes.

	Pregnancy Duration	Preterm Birth	Birth Weight	Low Birth Weight	Small Size for Gestational Age
	B (95% CI)	Exp (B) (95%CI)	B (95% CI)	Exp (B) (95%CI)	Exp (B) (95%CI)
HRQoL in early pregnancy					
PCS score	0.05 (0.00, 0.10)	0.96 (0.85, 1.08)	−4.57 (−19.55, 10.42)	1.07 (0.94, 1.23)	1.05 (0.96, 1.16)
MCS score	0.01 (−0.03, 0.06)	0.90 (0.82, 1.00)	7.90 (−5.62, 21.42)	0.96 (0.86, 1.08)	1.02 (0.94, 1.10)
HRQoL in mid-pregnancy					
PCS score	0.004 (−0.001, 0.008)	0.99 (0.98, 1.00)	−0.45 (−1.92, 1.03)	1.00 (0.99, 1.01)	1.00 (0.99, 1.01)
MCS score	−0.002 (−0.007, 0.002)	1.00 (0.99, 1.02)	0.44 (−1.92, 1.05)	1.00 (0.99, 1.02)	1.00 (0.99, 1.01)
HRQoL in late pregnancy					
PCS score	0.08 (0.03, 0.13)	0.90 (0.79, 1.03)	−18.10 (−33.43, −2.78)	1.01 (0.88, 1.17)	1.20 (1.08, 1.33) *
MCS score	0.007 (−0.04, 0.05)	0.96 (0.85, 1.09)	14.06 (0.01, 28.10)	0.96 (0.85, 1.09)	0.93 (0.85, 1.01)

Regarding physical and mental component summary scores, one-unit change in the regression model is 10 points of the original score. Values in this table are values of coefficient B, exp (B) with 95% CI (confidence interval). Values not present in this table are coefficient B, exp (B) with 95% CI (confidence interval) of covariates. One cell corresponds to one full model adjusted by covariates including maternal age at enrolment, gestational age at enrolment, parity, maternal educational level, ethnic background, body mass index at enrolment, and maternal smoking in each gestational period. * Asterisks indicate statistical significance based on the Bonferroni corrected *p* value (*p* < 0.008).

**Table 3:** Differences of birth outcomes between subgroups with very high and very low scores of physical and mental component summary (PCS/MCS) in each gestational period.

	Pregnancy Duration			Preterm Birth			Birth Weight			Low Birth Weight			Small for Gestational Age		
	Mean (SD)	*p* value	effect size	Yes (%)	No (%)	*p* value	Mean (SD)	*p* value	effect size	Yes (%)	No (%)	*p* value	Yes (%)	No (%)	*p* value
PCS score in early pregnancy															
<10th (n = 634)	39.8 (1.8)	0.18	0.06	32 (5.0)	602 (95.0)	0.60	3425 (559)	0.81	0.01	24 (3.8)	610 (96.2)	0.42	56 (8.8)	578 (91.2)	0.70
>90th (n = 686)	39.9 (1.8)			30 (4.4)	656 (95.6)		3418 (551)			33 (4.8)	653 (95.2)		65 (9.5)	621 (90.5)	
MCS score in early pregnancy															
<10th (n = 633)	39.8 (1.8)	0.31	0.06	40 (6.3)	593 (93.7)	0.55	**3351 (550)**	**0.005**	**0.15**	36 (5.7)	597 (94.3)	0.81	68 (10.7)	565 (89.3)	0.46
>90th (n = 640)	39.9 (1.8)			35 (5.5)	605 (94.5)		**3440 (582)**			34 (5.3)	606 (94.7)		60 (9.4)	580 (90.6)	
PCS score in mid-pregnancy															
<10th (n = 621)	39.8 (1.7)	0.01	0.12	38 (6.1)	583 (93.9)	0.03	3444 (555)	0.71	0.02	27 (4.3)	594 (95.7)	0.47	53 (8.5)	568 (91.5)	0.61
>90th (n = 622)	40.0 (1.6)			22 (3.5)	600 (90.5)		3433 (513)			22 (3.5)	600 (96.5)		48 (7.7)	574 (92.3)	
MCS score in mid-pregnancy															
<10th (n = 621)	39.8 (1.8)	0.08	0.11	40 (6.4)	581 (93.6)	0.17	**3376 (546)**	**0.001**	**0.18**	27 (4.3)	594 (95.7)	0.23	62 (10.0)	559 (90.0)	0.04
>90th (n = 631)	40.0 (1.5)			29 (4.6)	602 (95.4)		**3474 (518)**			19 (3.0)	612 (97.0)		42 (6.7)	589 (93.3)	
PCS score in late-pregnancy															
<10th (n = 604)	39.7 (1.6)	0.01	0.18	36 (6.0)	568 (94.0)	0.62	3480 (564)	0.01	0.15	27 (4.5)	577 (95.5)	0.79	**45 (7.5)**	**559 (92.5)**	**0.005**
>90th (n = 608)	40.0 (1.7)			32 (5.3)	576 (94.7)		3397 (563)			30 (4.7)	578 (95.1)		**75 (12.3)**	**533 (87.7)**	
MCS score in late-pregnancy															
<10th (n = 604)	39.9 (1.7)	0.07	0.06	35 (5.8)	569 (94.2)	0.08	**3344 (542)**	**<0.001**	**0.24**	30 (5.0)	574 (95.0)	0.15	**74 (12.3)**	**530 (87.7)**	**0.001**
>90th (n = 605)	40.0 (1.5)			22 (3.6)	583 (96.4)		**3474 (524)**			20 (3.3)	585 (96.7)		**39 (6.4)**	**566 (93.6)**	

The bold print indicates the statistical significance according to the Bonferroni-corrected *p* value (i.e., *p* < 0.008).

## Supplementary Materials

The following are available online at https://www.mdpi.com/1660-4601/16/21/4243/s1, Table S1: Non-response analysis (N = 9778); Table S2: Associations between PCS/MCS scores and birth weight, SGA in the subgroups reporting very high (>90th percentile) and very low (<10th percentile) scores, adjusted by confounders; Table S3: Associations between PCS/MCS in early, mid-, late pregnancy and offspring length and head circumference at birth adjusted by potential confounders; Table S4: Differences in offspring length and head circumference at birth between subgroups of women with very high and very low PCS/MCS scores; Table S5: Associations between PCS/MCS scores and offspring length, head circumference at birth among subgroups of women with very high (> 90th percentile) and very low (<10th percentile) PCS/MCS scores.
