# Supplementary material for: Associations between Maternal Health-Related Quality of Life during Pregnancy and Birth Outcomes: The Generation R Study
Source: Int J Environ Res Public Health. 2019 Nov 1;16(21):4243. doi: 10.3390/ijerph16214243 (PMC6862207; doi:10.3390/ijerph16214243)
Supplement: Supplementary file 1 [file ijerph-16-04243-s001.pdf]

International Journal of
Environmental Research and Public Health

MDPI

Supplementary Table S1. Non-response analysis (N=9778)

Variables	Values *		*p* value
	Population included in the analysis (n = 6334)	Population excluded from the analysis (n = 3444)	
Maternal age at enrolment (years), mean (SD)	29.9 (5.2)	29.8 (5.8)	0.28
Gestational age at enrolment (weeks), mean (SD)	15.0 (4.0)	16.0 (4.7)	<0.001
Parity, number (%)			<0.001
Nulliparous	3679 (58.2)	1499 (48.9)	
Multiparous	2642 (41.8)	1569 (51.1)	
Missing	*13*	*376*	
Educational level, number (%)			<0.001
High education	1563 (25.1)	442 (19.0)	
Mid-high	1234 (19.8)	421 (18.1)	
Mid-low	1929 (30.9)	698 (30.0)	
Low	1507 (24.2)	763 (32.8)	
Missing	*101*	*1120*	
Ethnic background, number (%)			<0.001
Dutch	3375 (53.5)	1170 (42.1)	
Non-Dutch, Western	552 (8.8)	224 (8.1)	
Non-Dutch, non-Western	2378 (37.7)	1385 (49.8)	
Missing	*29*	*665*	
Body Mass Index at enrolment, mean (SD)	24.7 (4.5)	25.5 (4.7)	<0.001
Missing	29		
Maternal smoking in early pregnancy, number (%)			0.03
Never smoking	4738 (75.9)	1005 (74.6)	
Stopped smoking when the pregnancy was known	761 (12.2)	148 (11.0)	
Continuing smoking in pregnancy	746 (11.9)	195 (14.5)	
Missing	*89*	*2096*	
Birth outcomes			
Pregnancy duration (weeks), number (%)	39.9 (1.7)	39.5 (2.3)	<0.001
Preterm birth (yes), number (%)	331 (5.2)	292 (9.1)	<0.001
Birth weight (grams), mean (SD)	3428 (558)	3334 (605)	<0.001
Low birth weight (yes), number (%)	290 (4.6)	228 (7.1)	<0.001
Small for gestational age (yes), number (%)	604 (9.5)	353 (11.3)	<0.001

* Values are means, standard deviations, numbers and percentages.

Supplementary Table S2. Associations between PCS/MCS scores and birth weight, SGA (Small for Gestational Age) in the subgroups reporting very high (> 90th percentile) and very low (<10th percentile) scores, adjusted by confounders.

	Birth Weight, Grams		SGA	
	B [95% CI]	*p* value	OR [95% CI]	*p* value
PCS in late pregnancy (very high vs. very low)	NA	NA	**1.82 [1.18, 2.79]**	**0.006**
MCS in early pregnancy (very high vs. very low)	44.7 [−20.2, 109.5]	0.18	NA	NA
MCS in mid-pregnancy (very high vs. very low)	12.8 [−55.2, 80.7]	0.71	NA	NA
MCS in late pregnancy (very high vs. very low)	45.4 [−24.0, 114.9]	0.20	0.69 [0.43, 1.11]	0.13

Values in this table are values of coefficient B, exp (B) with 95% CI (confidence interval). Each cell represents one full model adjusted by potential confounders including maternal age at enrolment,

gestational age at enrolment, parity, maternal educational level, ethnic background, Body Mass Index at enrolment, and maternal smoking in each gestational period.

Supplementary Table S3. Associations between PCS/MCS in early, mid-, late pregnancy and offspring length and head circumference at birth adjusted by potential confounders.

	**Offspring Length, cm**		**Offspring Head Circumference, cm**	
	B [95% CI]	*p* value	B [95% CI]	*p* value
		HRQoL in early pregnancy		
PCS	0.000 [−0.009, 0.009]	0.97	−0.004 [−0.011, 0.003]	0.27
MCS	0.009 [0.001, 0.017]	0.03	0.002 [−0.004, 0.009]	0.53
		HRQoL in mid-pregnancy		
PCS	−0.001 [−0.009, 0.008]	0.88	−0.004 [−0.011, 0.004]	0.32
MCS	0.008 [−0.001, 0.017]	0.09	−0.001 [−0.008, 0.006]	0.83
		HRQoL in late pregnancy		
PCS	0.002 [−0.007, 0.011]	0.69	−0.003 [−0.011, 0.005]	0.49
MCS	0.005 [−0.004, 0.013]	0.26	0.000 [−0.007, 0.008]	0.95

Values in this table are values of coefficient B with 95% CI (confidence interval). Each cell represents one full model adjusted by covariates including maternal age at enrolment, gestational age at enrolment, parity, maternal educational level, ethnic background, Body Mass Index at enrolment, and maternal smoking in each gestational period. cm: centimeter.

Supplementary Table S4. Differences in offspring length and head circumference at birth between subgroups of women with very high and very low PCS/MCS scores.

	**Offspring Length at Birth, cm**		**Offspring Head Circumference at Birth, cm**	
	Mean (SD)	*p* value	Mean (SD)	*p* value
		PCS in early pregnancy		
<10th	51.0 (2.8)	0.79	35.2 (2.3)	0.43
>90th	50.9 (2.8)		35.1 (2.2)	
		MCS in early pregnancy		
<10th	50.7 (2.9)	0.005	34.9 (2.4)	0.26
>90th	51.2 (2.7)		35.1 (2.4)	
		PCS in mid-pregnancy		
<10th	51.0 (2.9)	0.98	35.0 (2.3)	0.59
>90th	51.0 (2.9)		35.1 (2.4)	
		MCS in mid-pregnancy		
<10th	50.6 (2.9)	0.001	34.8 (2.6)	0.09
>90th	51.2 (3.0)		35.1 (2.3)	
		PCS in late pregnancy		
<10th	51.0 (2.9)	0.76	35.1 (2.5)	0.47
>90th	51.1 (3.0)		35.0 (2.4)	
		MCS in late pregnancy		
<10th	50.6 (2.7)	0.001	34.7 (2.4)	0.004
>90th	51.1 (2.9)		35.2 (2.4)	

Supplementary Table S5. Associations between PCS/MCS scores and offspring length, head circumference at birth among subgroups of women with very high (>90th percentile) and very low (<10th percentile) PCS/MCS scores.

	**Offspring Length at Birth, cm**		**Offspring Head Circumference at Birth, cm**	
	B [95% CI]	*p* value	B [95% CI]	*p* value
MCS in early pregnancy (very high vs. very low)	0.24 [−0.12, 0.61]	0.19	NA	NA
MCS in mid-pregnancy (very high vs. very low)	0.26 [−0.17, 0.69]	0.24	NA	NA

MCS in late pregnancy (very high vs. very low)	0.29 [−0.13, 0.71]	0.17	0.28 [−0.08, 0.64]	0.12

Values in this table are values of coefficient B with 95% CI (confidence interval). One cell is corresponding to one full model adjusted by covariates including maternal age at enrolment, gestational age at enrolment, parity, maternal educational level, ethnic background, Body Mass Index at enrolment, and maternal smoking in each gestational period.

© 2019 by the authors. Licensee MDPI, Basel, Switzerland. This article is an open access article distributed under the terms and conditions of the Creative Commons Attribution (CC BY) license (http://creativecommons.org/licenses/by/4.0/).
